# Versatile Ultrasound-Compatible Microfluidic Platform for In Vitro Microvasculature Flow Research and Imaging Optimization

**DOI:** 10.1021/acsomega.3c05849

**Published:** 2023-12-05

**Authors:** Tamar Mano, Tal Grutman, Tali Ilovitsh

**Affiliations:** † Department of Biomedical Engineering, Tel Aviv University, Tel Aviv 6997801, Israel; ‡ The Sagol School of Neuroscience, Tel Aviv University, Tel Aviv 6997801, Israel

## Abstract

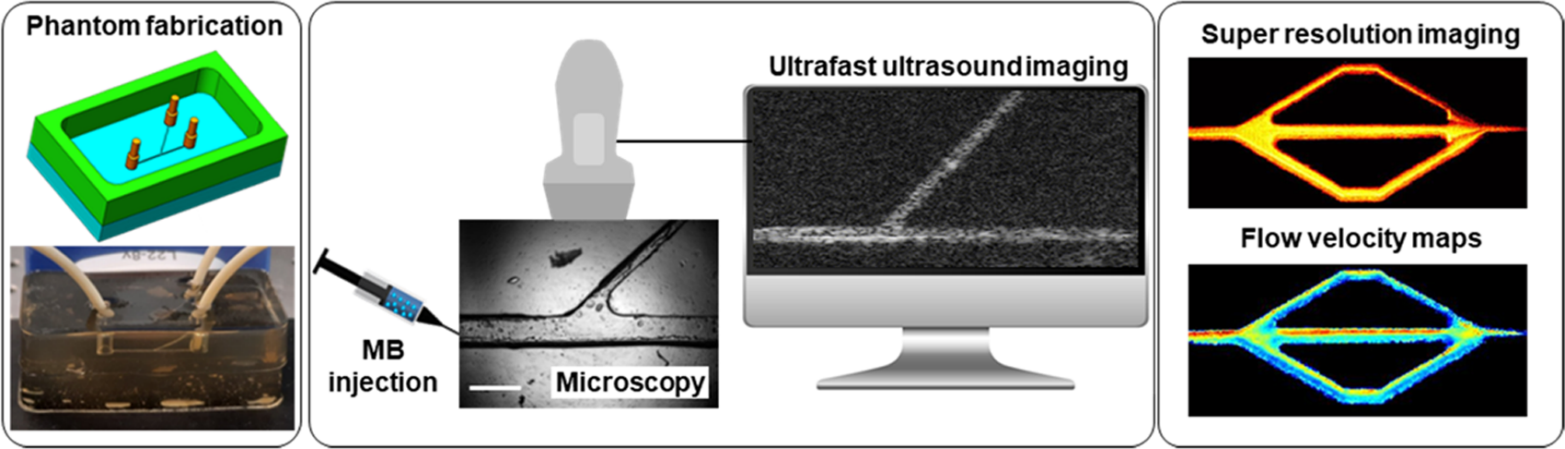

Ultrasound localization microscopy (ULM) enables the creation of super-resolved images and velocity maps by localizing and tracking microbubble contrast agents through a vascular network over thousands of frames of ultrafast plane wave images. However, a significant challenge lies in developing ultrasound-compatible microvasculature phantoms to investigate microbubble flow and distribution in controlled environments. In this study, we introduce a new class of gelatin-based microfluidic-inspired phantoms uniquely tailored for ULM studies. These devices allow for the creation of complex and reproducible microvascular networks featuring channel diameters as small as 100 μm. Our experiments focused on microbubble behavior under ULM conditions within bifurcating and converging vessel phantoms. We evaluated the impact of bifurcation angles (25, 45, and 55°) and flow rates (0.01, 0.02, and 0.03 mL/min) on the acquisition time of branching channels. Additionally, we explored the saturation time effect of narrow channels branching off larger ones. Significantly longer acquisition times were observed for the narrow vessels, with an average increase of 72% when a 100 μm channel branched off from a 300 μm channel and an average increase of 90% for a 200 μm channel branching off from a 500 μm channel. The robustness of our fabrication method is demonstrated through the creation of two trifurcating microfluidic phantoms, including one that converges back into a single channel, a configuration that cannot be achieved through traditional methods. This new class of ULM phantoms serves as a versatile platform for noninvasively studying complex flow patterns using ultrasound imaging, unlocking new possibilities for in vitro microvasculature research and imaging optimization.

## Introduction

Ultrasound localization microscopy (ULM) is an ultrasound imaging technique used to overcome the diffraction limit and image microvascular flow deep within tissues. In ULM, contrast agents in the form of gaseous microbubbles (MBs) are injected into the bloodstream where they are individually localized and tracked over thousands of frames. Tracking of MBs at high frame rates through microvessels allows to reconstruct super-resolved images and velocity maps of vascular structures as small as 5 μm, an order of magnitude below the diffraction limit.^1−3^ ULM has been demonstrated in simulations, tissue-mimicking phantoms, and in vivo in different organs such as rat brains^4,5^ and kidneys.^3,6^ In vivo ULM is limited in the ability to compare different conditions and perform repetitive imaging of identical vascular networks for statistical purposes. In addition, the ground-truth vessel sizes, flow rate, and full configuration of the network are generally not known. It is known that MBs behave and oscillate differently in different sized vessels.^7,8^ Therefore, the creation of a blood-vessel-mimicking phantom for the ULM is of great interest.

Significant challenges exist in engineering complex tissue-mimicking vascular phantoms for ultrasound imaging applications. Due to their complexity, most ULM phantoms rely on single channels. The single channel can be placed within a water tank; however, the vessel boundaries reproduce a strong echo that obscures the MB signal and needs to be filtered out. Alternatively, the channel can be created by embedding a tube within an agarose- or gelatin-based mold, and after solidifying, the tube is pulled out and an empty channel is formed. Although useful, this technique is suitable mostly for single wide channels. Channels smaller than 200 μm are difficult to fabricate and handle due to the need to inject the MB suspension into the channel. Nevertheless, elaborate flow phantoms that contain branching channels are vital to the study of flow patterns by using ultrasound. In ultrasound imaging, an X shape phantom was created by crossing two single channels; however, since the channels were located one on top of the other, they are inherently unaligned in the elevation plane.^4^ A custom 3D printer was also developed for the creation of ultrasound flow phantoms, yet it is a costly solution that created vessels with diameters of 200 μm.^9,10^ CAD modeling was used to create a complex flow phantom using a polyvinyl alcohol cryogel as the material. This study was used to fabricate a patient-specific, complex flow model that may be used for flow imaging or super-resolution imaging in large blood vessels. However, it is not suitable for small vessels, and the fabrication method is complex.^11^ In a 3D super-resolution imaging study, a phantom was created by using two 200 μm cellulose tubes surrounded by paraffin wax gel to form two channels. These single channels can be imaged together when conducting 3D imaging but do not branch out or merge.^12^ A wire templating method was able to create capillary-scale bifurcating channels. This is a complex solution in which channels can only split into smaller channels, but cannot converge back into a larger channel.^13^ Microfluidic devices are yet another alternative that are a well-established platform for the study of flow using optical imaging. Recently, such devices were used to image MB oscillations following ultrasound excitation.^14^ These devices are typically composed of polymeric materials such as polydimethylsiloxane (PDMS), which highly attenuates ultrasound. Therefore, the use of microfluidic devices for ULM is limited due to the weak signal that echoes from isolated MBs. Many additional phantom studies were limited to the millimeter range in vessel size and complexity.^2,5,15,16^ Our aim was to develop a new class of ULM phantoms that contain complex blood-vessel-mimicking channels with full control over bifurcating vessel thickness, angle, and length to provide a manner to study ULM in a controlled environment and serve as a prerequisite step prior to in vivo experiments. We fabricated a gelatin based phantom using a two part machined mold with one side consisting of the negative side of the planned vessel network, with rods to create inlets and outlets for controlled flow. Gelatin is an ultrasound-compatible material with minimal attenuation. In addition, it fully cross-links after congelation. Therefore, the two parts were extracted and combined when the gelatin partially polymerized and the gelatin fully polymerized in the final assembly, to yield a one-piece bonded ULM phantom. This phantom was used here to study the physical properties of MB flow and distribution using ULM. First, we characterized the saturation time as a function of microbubble concentration. Next, we reconstructed velocity maps of phantoms with bifurcation angles of 25–55° at flow rates from 0.01 to 0.03 mL/min and explored the effect of the bifurcation angle and flow rate on the acquisition time. Acquisition time to full saturation of phantoms with vessel widths ranging from 500 to 100 μm was also assessed. Next, phantoms with background scatterers are introduced. Lastly, we demonstrate the robustness of the fabrication method by creating two trifurcating microfluidic-style phantoms.

## Materials and Methods

### Microvessel Phantom Fabrication

**Figure 1:**
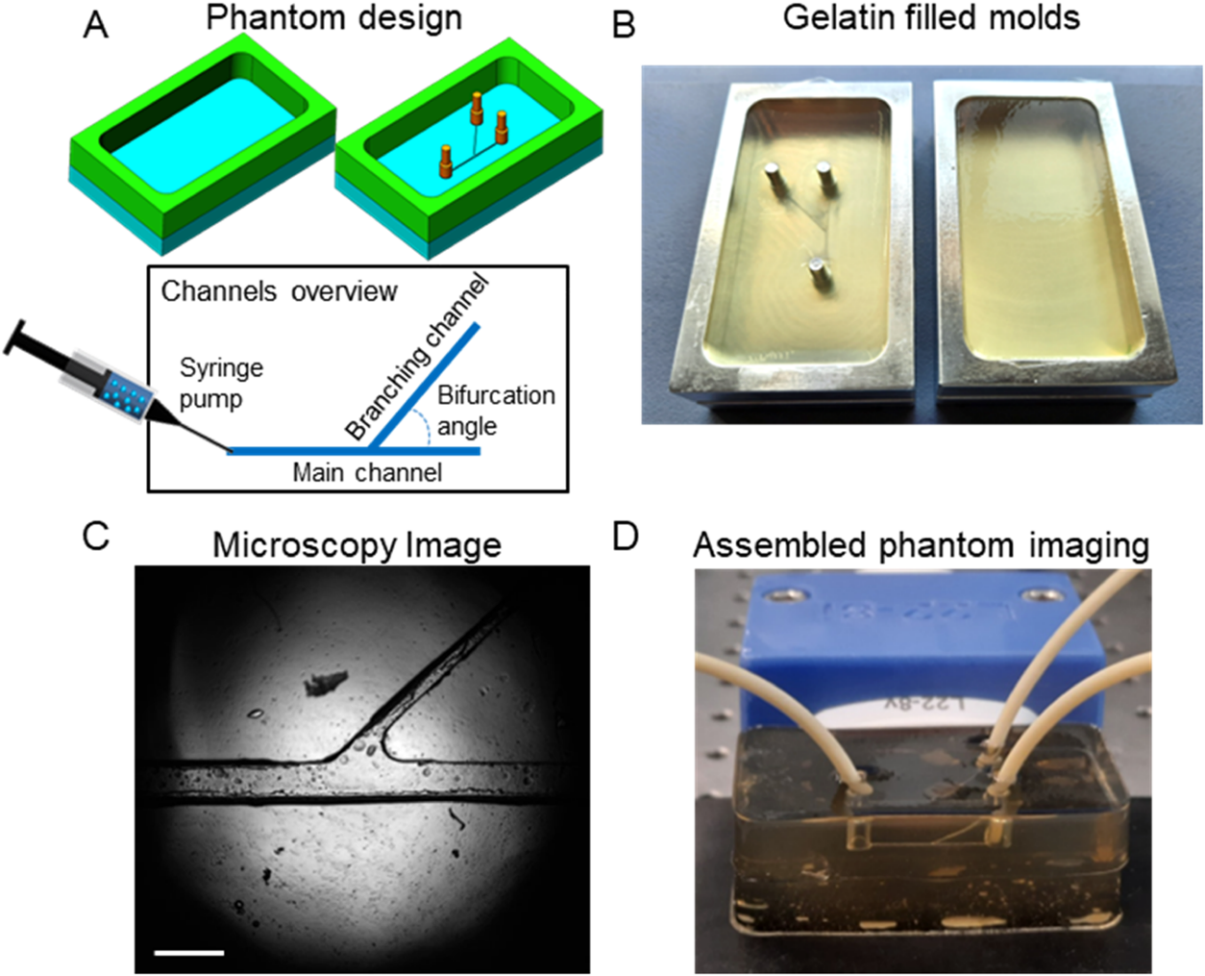
Microvessel phantom fabrication. (A) Schematic illustration of the two-part mold and overview of the network. (B) Gelatin poured into aluminum molds. (C) Microscopic image of gelatin-based channels; scale bar is 1 mm. (D) Assembled phantom with tubing connected to inlets and outlets, imaged with an L22–8v transducer.

The process used to fabricate gelatin phantoms with branching microfluidic channels is shown in Figure 1. The method is inspired by techniques used in tissue-engineering studies.^17,18^ A two-part aluminum mold was fabricated by a computerized numerical control (CNC) machine. Each part consisted of a base and walls connected by screws. The base of the first part consisted of a protruded negative of the desired microfluidic channel with rods that will create inlets to the phantom. The second base was smooth (Figure 1A). The process allowed full control over the main and branching vessels thicknesses and the bifurcation angle. After fabrication of the aluminum molds, the channel width was measured at three points in the beginning, middle, and end of the channel using a digital caliper to ensure a maximal deviation of 0.01 mm for all channel widths. Gelatin powder (G9382, Sigma-Aldrich) was mixed with deionized water to a 10% solution at ambient temperature and heated until all powder was completely dissolved. The solution was then poured into the molds and allowed to cool for 2 h at room temperature (Figure 1B). The partially cross-linked gelatin was then demolded, carefully assembled together, with the channel positioned in the middle of the assembly, and placed at 4 °C for 6 h to fully cross-link. For the experiment that contained acoustic scatters in the phantom, background scatterers were achieved by allowing the heated gelatin to cool to room temperature with a magnetic stirrer and adding 0.5% dietary fiber powder composed of wheat dextrin to the solution. When the fibers were fully mixed, the resulting solution was poured into the molds.

Each assembled phantom was lightly sprayed with ethanol to discourage mold growth and stored in a closed box at 4 °C for up to 5 days. Although after 5 days there was no visible mold growth on the phantom, we suggest using the phantom within this time period to ensure structural integrity and avoid the possibility of unseen mold growth in its beginning stages. In our research and all displayed experiments, we used only phantoms created on the day before each experiment.

Seven different phantom configurations were created. Each had a different base that corresponded to the desired branching pattern. All configurations had a channel height of 300 μm. The bifurcating phantoms consisted of a main channel and a branching channel that split off from the main channel. The first set of three phantoms consisted of channel widths of 300 μm with branching channels at 25, 45, and 55° angles. A fourth configuration had a main channel width of 500 μm and a branching channel width of 200 μm with a 45° bifurcation. The fifth configuration had a main channel width of 300 μm and a branching channel width of 100 μm with a 45° bifurcation. The two additional phantoms consisted of trifurcating channels at angles of 30° and 300 μm channel widths. The first included a main channel that split into three equally sized channels, each reaching a separate outlet. In the second, the channel split into three equally sized channels and then converged back into one channel, similar to classical microfluidic chips. Prior to each experiment, the phantom inlets were connected with tubing to a 2.5 mL syringe filled with diluted MB solution. The syringe was placed on a programmable flow-inducing pump (GenieTouch, Kent Scientific, Torrington) set at flow rates of 0.01, 0.02, and 0.03 mL/min. The flow rates in the various phantoms span a velocity range of 1–17 mm/s, covering the span of velocities detected by ULM in vivo studies.^4^

### Microbubble Preparation

MBs were composed of a phospholipid shell and a perfluorobutane (C_4_F_10_) gas core and prepared as reported in previous studies.^19−21^ Before use, the MB vials were shaken for 45 s in a vial shaker and purified via centrifugation to remove MBs with radii smaller than 0.5 μm. The size and concentration of MBs were measured using a particle counter system (Accusizer FXNano, Particle Sizing Systems, Entegris, MA). The MBs were used within 3 h of their preparation. The size distribution and concentration varied by less than 10% between measurements. The MBs were diluted with phosphate buffer saline in a 2.5 mL syringe to concentrations of 1.6 × 10^6^, 6.4 × 10^6^, and 6.4 × 10^7^ MBs/mL to test the acquisition time to full saturation as a function of MB concentration. For all other experiments, MBs were diluted to 6.4 × 10^6^ MBs/mL.

### Ultrasound Acquisition

A high-frequency transducer L22–8 (Kolo Medical) controlled by a programmable ultrasound system (Vantage 256, Verasonics, WA) was used for ultrafast imaging of MBs using a custom contrast pulse sequence (CPS) written in MATLAB (version 2020a, MathWorks, Natick, MA). A center frequency of 10 MHz was transmitted, and the second harmonic frequency of 20 MHz was received, both within the bandwidth of the transducer. Imaging was performed at a mechanical index (MI) of 0.14. The MI, a parameter that determines the likelihood of creating mechanical damage within the tissue as a result of US application, is defined as the peak negative pressure (PNP) divided by the square root of frequency. For imaging applications, the food and drug administration (FDA) limits the MI to a value below 1.9.^22^ The MI was calibrated by using a needle hydrophone (NH0200, Precision Acoustics, U.K.) in a degassed water tank. Frames were coherently compounded with angles at −5, 0, and +5°, where at each angle three successive pressure waves were transmitted using a CPS with a summed overall amplitude of zero (0.5, −1, 0.5) to take advantage of the nonlinear scattering response of MBs and reduce the response of linear scatters.^23^ Phantoms with acoustic scatterers were also imaged using the B-mode in which plane waves were coherently compounded with angles at −5, 0, and +5°. Data were recorded in continuous 1500 frame blocks at a frame rate of 250 Hz. Velocity maps were reconstructed using a total of 9000 frames, and saturation curves were calculated over 3000 frames. MB concentration and vessel width experiments were performed at a flow rate of 0.02 mL/min.

### ULM Image Processing and Data Processing

The ULM algorithm consisted of up-sampling and interpolating each original frame to 2× the original using the pretrained Fast Super-Resolution Convolutional Neural Network (FSRCNN).^24^ Next, MB signals were enhanced using singular value decomposition filtered by removing the first two singular values^25^ and applying a second-order high pass filter. Peak detection and localization were performed using adaptive filtering and calculation of a weighted average on neighboring pixel intensities. Localized PSF tracking was achieved by calculating the minimum distance between PSFs in consecutive frames, and velocity was computed as the displacement between consecutive frames. Only MBs that could be tracked for more than 10 consecutive frames were included in the velocity calculations.

Expected velocity within the channels was calculated using Euler’s simplified conservation of mass with known cross sections and a known flow rate at the input of the phantom, according to
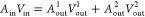
1where *A* is the area of the rectangular cross section and *V* is the velocity within the channel of the flow at the input and outputs of the phantom. In the phantom with equal channel cross sections for the main and branching channels, *V*_out_^1^ = *V*_out_^2^ from symmetry.

Saturation time curves were created by setting a range of interest (ROI) of 25 × 25 pixels immediately after or before the bifurcation in a super-resolved image. The localizations of MBs within the channel starting at time 0 until full saturation of the channel were then observed. Full saturation occurs when a MB localization event occurs in all of the pixels within the vessel passing through the ROI. The curves were normalized by their maximal value and fit to an exponential function to calculate the time to 63% of full saturation, defined as τ. In an exponentially increasing function, τ is described as the time to 63% of the function’s final value. We used this definition to quantitatively characterize the increase in the saturation curves. Statistical analyses were performed by using Prism9 software (GraphPad Software Inc.). The results are presented as the mean ± SD. Statistical tests are reported in the relevant captions. *P* values less than 0.05 were considered significant.

## Results

### MB Concentration Optimization Results

**Figure 2:**
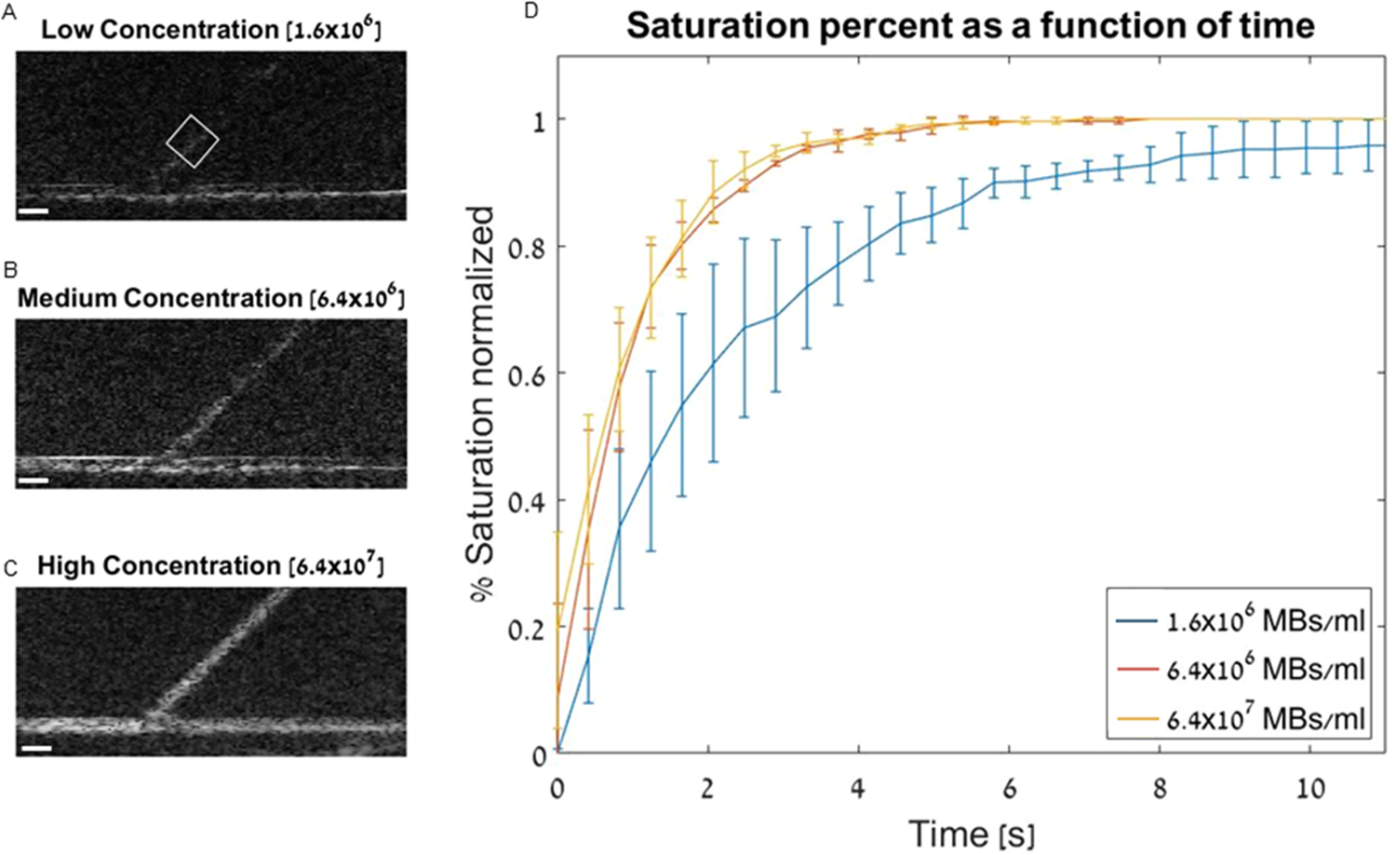
MB concentration optimization in a bifurcating vessel phantom with a channel diameter and height of 300 μm and a bifurcation angle of 45°. (A–C) Representative images from each MB concentration data set, including a white rectangle 25 × 25 pixel ROI used for all calculations. All scale bars are 1 mm. MB concentration: (A) 1.6 × 10^6^ MBs/mL; (B) 6.4 × 10^6^ MBs/mL; and (C) 6.4 × 10^7^ MBs/mL. (D) Normalized saturation percent of the ROI over time for the three MB concentrations. *R*^2^ > 0.98 for all fit curves used to calculate τ. All experiments were performed in triplicate, and all data are plotted as mean ± SD.

Initial experiments were performed by using a 300 μm vessel that branched into two channels of 300 μm at a splitting angle of 45°. First, the optimal MB concentration for phantom imaging was identified by assessing the image saturation curve over time for three MB concentrations (Figure 2). Saturation was calculated in a 25 × 25 pixel range immediately after the bifurcation (Figure 2A). Full saturation was achieved when localized MB events were detected in each pixel within the vessel passing through the window (white rectangle, Figure 2A). The characteristic time to 63% saturation (τ) was calculated for each curve fitted to an exponential function. The lowest concentration evaluated, 1.6 × 10^6^ MBs/mL, yielded significantly longer times to saturation of the branching channel. The low-concentration (1.6 × 10^6^ MB/mL) τ value of 2.15 ± 0.85 s was found to be significantly higher (*P* < 0.05) than both the high- and medium-concentration values. The medium concentration (6.4 × 10^6^ MB/mL) and high concentration (6.4 × 10^7^ MB/mL) yielded similar τ values of 0.94 ± 0.23 and 0.83 ± 0.25 s, respectively (*P* > 0.05, Figure 2). The high MB concentration was such that individual MBs were overlapping, which lowered the MB tracking accuracy and affected the final super-resolved images. The medium concentration of 6.4 × 10^6^ MB/mL yielded an optimal saturation time without compromising the algorithm’s tracking accuracy and hence was used in all following experiments.

### Velocity Estimation as a Function of the Bifurcation Angle

**Figure 3:**
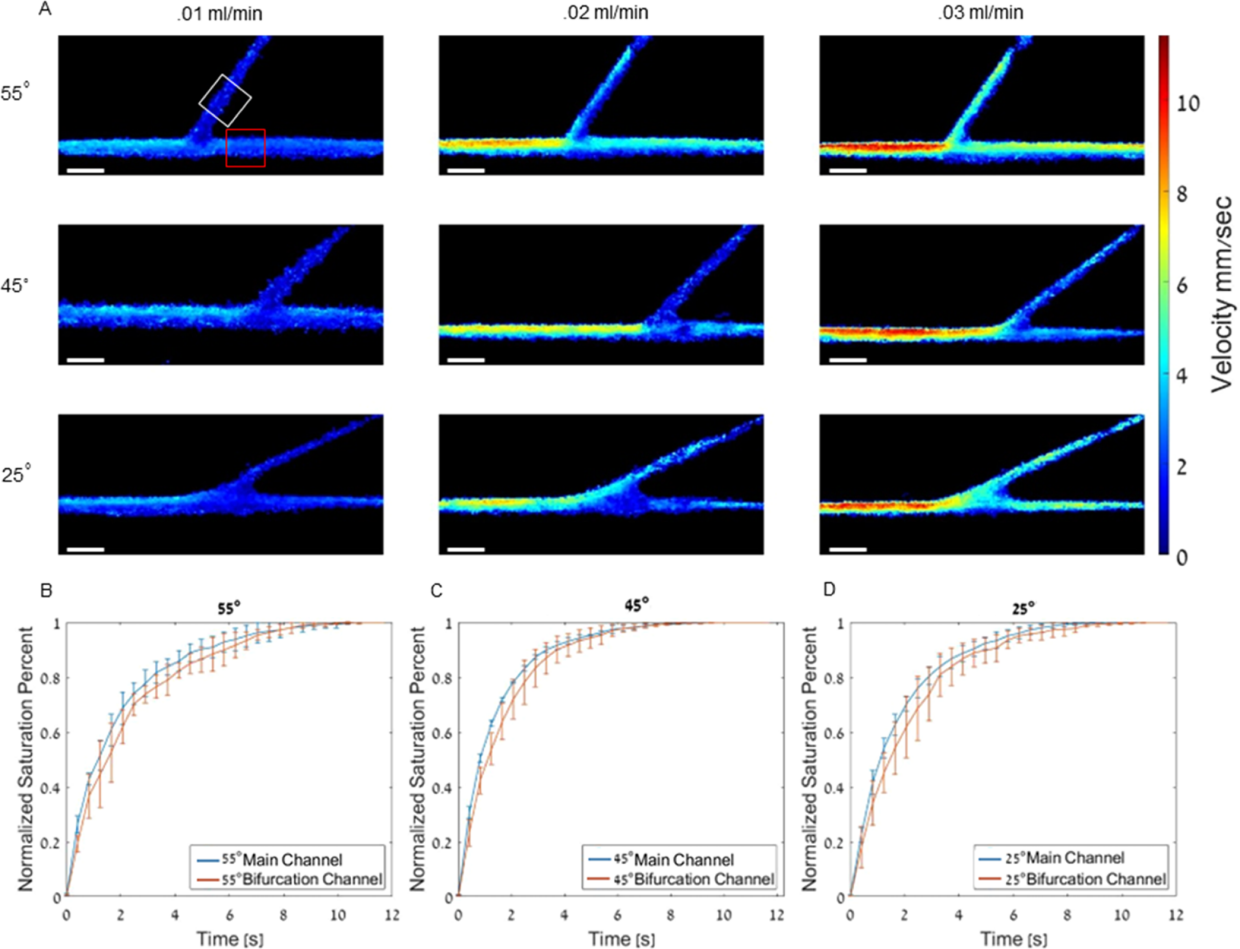
Velocity maps and saturation curves as a function of the bifurcation angle. (A) Velocity maps of phantoms with bifurcation angles of 55, 45, and 25° with flow rates of 0.01, 0.02, and 0.03 mL/min. All scale bars are 1 mm. (B–D) Saturation curve for the main channel and branching channel with bifurcation angles of (B) 55°, (C) 45°, and (D) 25°. *P* value > 0.05 for all groups. *R*^2^ > 0.98 for all fit curves used to calculate τ. All experiments were performed in triplicate, and all data are plotted as the mean ± SD.

The effect of bifurcation angle and velocity on full channel saturation time was evaluated next. In these phantoms, both the main and branched channels had a diameter of 300 μm, while the branching angle was 25, 45, or 55°. Flow velocity profiles were detected by tracking the flowing MBs in the phantoms for three flow velocities of 0.01, 0.02, and 0.03 mL/min (Figure 3). The saturation time curves were calculated for the main and branching channel to evaluate the effect of bifurcation angle on saturation time of the branching channel vs the main channel (red and white squares in Figure 3A). The 55° bifurcation yielded a τ value of 1.84 ± 0.28 s for the main channel and 2.20 ± 0.30 s for the branching channel. The 45° bifurcation yielded a τ value of 1.30 ± 0.01 s for the main channel and 1.62 ± 0.32 s for the branching channel. The 25° bifurcation yielded a τ value of 1.72 ± 0.18 s for the main channel and 2.12 ± 0.52 s for the branching channel. No significant difference was found for τ values of the main and branching channel for the tested bifurcation angles and flow rates (*P* > 0.05).

**Figure 4:**
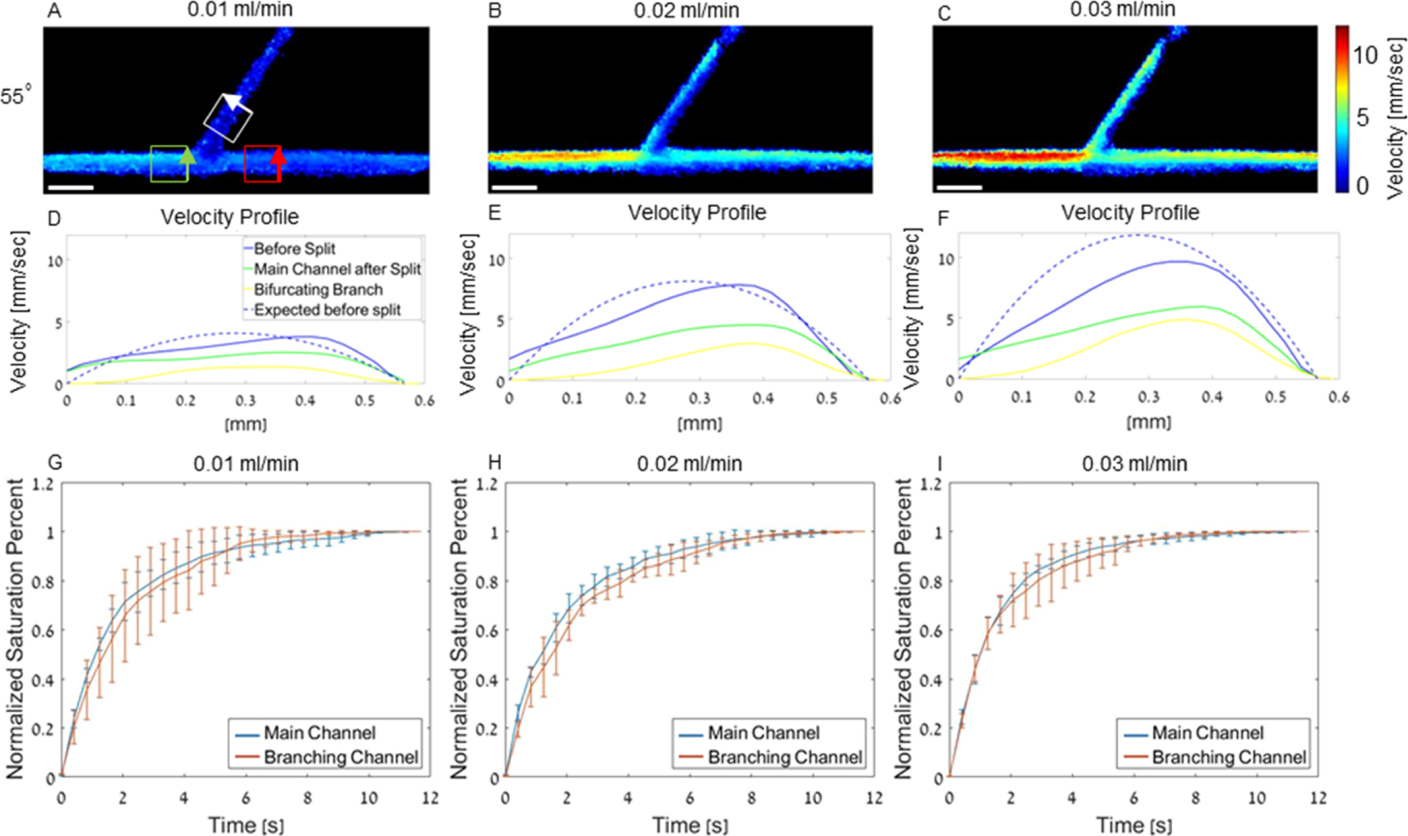
Velocity profiles and saturation curves as a function of the flow rate. (A–C) Velocity maps of channels with a 55° bifurcation and flow rates of 0.01, 0.02, and 0.03 mL/min. Arrows on the boxes in panel (A) indicate the direction corresponding to the flow profiles in panels (D–F). All scale bars are 1 mm. (D–F) Velocity profiles showing the expected velocity of the main channel before the bifurcation, calculated velocity of the main channel before the bifurcation, and of both channels after the bifurcation. (G) Saturation curve for the main and branching channel at 0.01 mL/min. (H) Saturation curve for the main and branching channel at 0.02 mL/min. (I) Saturation time curve of the main and branching channel at 0.03 mL/min. No significant difference was found, *P* value > 0.05 for all flow rates. *R*^2^ > 0.98 for all fit curves used to calculate τ. All experiments were performed in triplicate, and all data are plotted as ± SD.

Velocity profiles were calculated for the main channel before the bifurcation and both the main and branching channel after the bifurcation (Figure 4). Measured velocity profiles were calculated by averaging 25 successive channel cross sections in the ROI in the white, red, and green boxes (Figure 4A). The arrows on the boxes indicate the direction corresponding to the flow profiles in Figure 4D–F. Expected velocity of the main channel was calculated using the known flow rate and area of the channels, based on eq 1. The laminar flow in the channels forms the parabolic profiles (Figure 4D–F). Next, the effect of the input flow rate on the saturation of the branching channel compared to the main channel after the bifurcation was evaluated (Figure 4G–I). Saturation time curves of the main and bifurcating channel were compared for each flow rate displayed. At 0.01 mL/min, τ = 1.75 ± 0.30 s for the main channel and 2.13 ± 1.08 s for the branching channel. At 0.02 mL/min, τ = 1.84 ± 0.28 s for the main channel and 2.20 ± 0.30 for the branching channel. At 0.03 mL/min, τ = 1.50 ± 0.20 s for the main channel and 1.70 ± 0.58 for the branching channel (*P* > 0.05).

### Effect of Channel Widths

**Figure 5:**
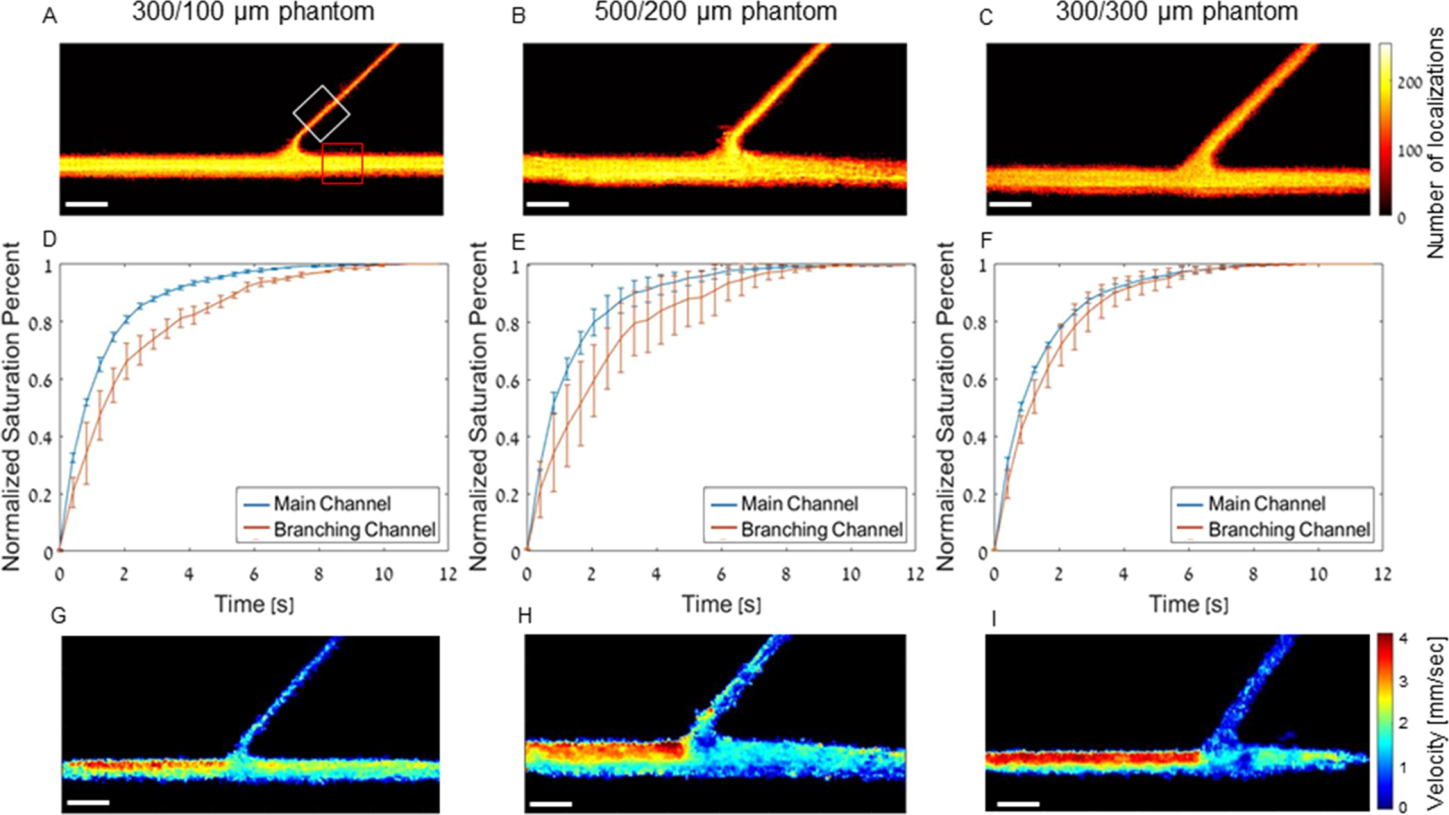
Super-resolved images of phantoms with varying channel widths and saturation time curves. (A) Super-resolved image of phantom with a 300 μm main channel and 100 μm branching channel. (B) Super-resolved image of phantom with a 500 μm main channel and 200 μm branching channel. (C) Super-resolved image of phantom with 300 μm main and branching channel. All scale bars are 1 mm. (D) Saturation curve for the main and branching channel in the 300/100 μm phantom, *P* value > 0.05. (E) Saturation curve for the main and branching channel in the 500/200 μm phantom, *P* value > 0.05. (F) Saturation curve for the branching channel in the 300/300 μm phantom, *P* value < 0.05. *R*^2^ > 0.98 for all fit curves used to calculate τ. (G) Velocity map of the 300/100 μm phantom at a flow rate of 0.01 mL/min. (H) Velocity map of the 500/200 μm phantom at a flow rate of 0.02 mL/min. (I) Velocity map of the 300/300 μm phantom at a flow rate of 0.01 mL/min. All experiments were performed in triplicate, and all data are plotted as ±SD.

The effect of the vessel diameter was evaluated. As often occurs in vivo, we focused on assessing the behavior of MBs flowing from a large channel into a smaller channel. The flow rate in all data sets was 0.02 mL/min. Three phantoms were evaluated: (1) main channel of 300 μm and a branching channel of 100 μm; (2) main channel of 500 μm and a branching channel of 200 μm; and (3) main channel of 300 μm and a branching channel of 300 μm. Their ULM images are shown in Figure 5A–C, respectively. The saturation curves of the 300 μm main channel (τ = 1.21 ± 0.05 s) and 100 μm branching channel (τ = 2.09 ± 0.33 s) have significantly different characteristic τ values when tested with a two-sample *t* test (Figure 5D, *P* < 0.05). The saturation curves for the 500 μm main channel (τ = 1.20 ± 0.07 s) and 200 μm branching channel (τ = 2.37 ± 0.70 s) also exhibit significantly different τ values (Figure 5E, *P* < 0.05). The phantom that consisted of equal main (τ = 1.30 ± 0.01 s) and branching (τ = 1.62 ± 0.32 s) channels of 300 μm diameter did not show difference in τ values (*P* > 0.05). This suggests that the branching into a smaller vessel is a main parameter that needs to be taken into account when conducting ULM imaging.

### Phantoms with Scatterers

**Figure 6:**
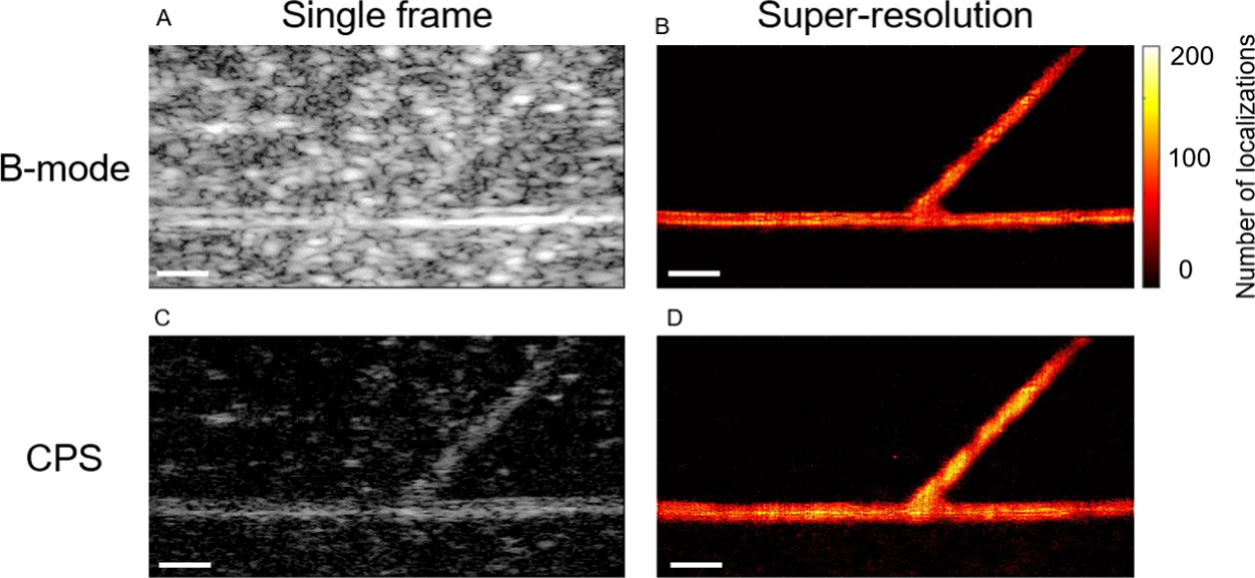
Super-resolved images using the B-mode and CPS with background scatterers. (A) Example image from the sequence using B-mode plane wave imaging. (B) Super-resolved image of the phantom using the B-mode data. (C) Example image from the sequence using CPS imaging. (D) Super-resolved image of the phantom using the CPS data. All scale bars are 1 mm.

To better establish the fabrication method of these phantoms and allow them to be relevant for a larger range of studies, we tested the ability to add background scatterers to better mimic soft tissue properties. Additionally, we compared B-mode (Figure 6A,B) and CPS (Figure 6C,D) acquisition sequences and our algorithm’s ability to localize bubbles using these different techniques. MB flow through phantoms can be observed in the B-mode and CPS in Supporting Video 1. Comparing Figure 6A,C highlights the ability of the CPS pulse sequence to remove a significant portion of the background scatter while retaining echoes from MBs flowing through the phantom. Our algorithm successfully localizes MBs using both pulse sequences; however, we note that the results are slightly noisier compared to the phantom without scatterers.

### Trifurcating Phantoms

**Figure 7:**
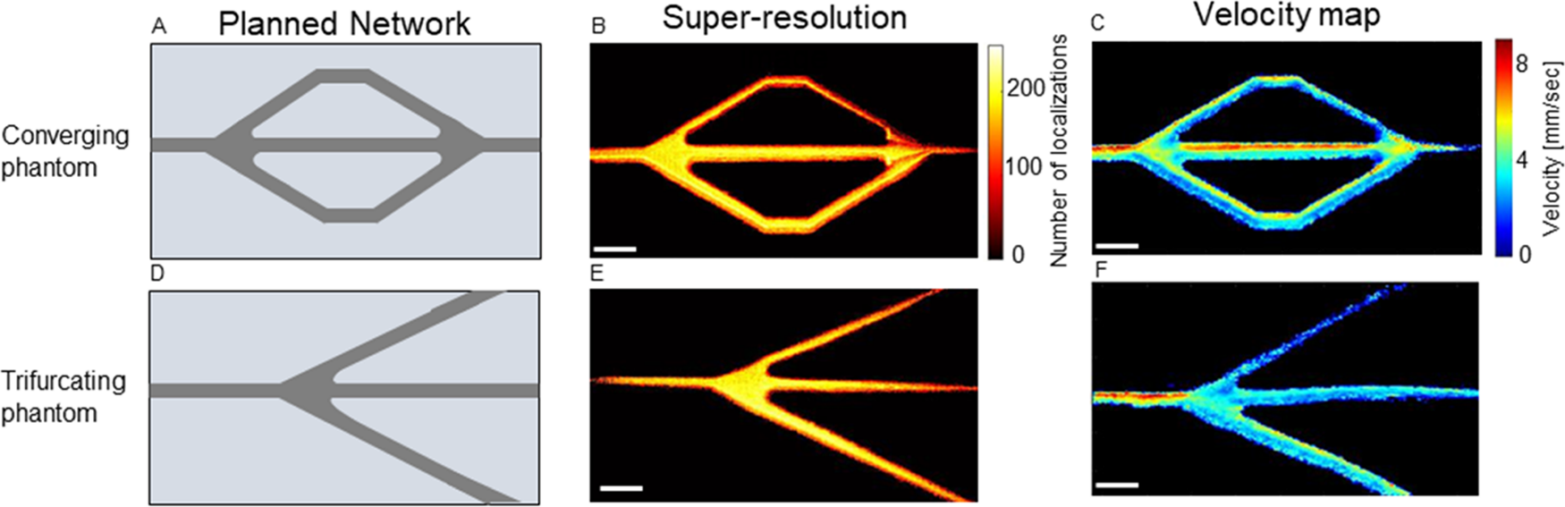
Super-resolved images and velocity maps of trifurcating phantoms. (A) Planned network for a microfluidic-style phantom with trifurcating channels that converge back to one main channel. (B) Super-resolved image of a converging phantom. (C) Velocity map of converging phantom at a flow rate of 0.02 mL/min. (D) Planned network for the trifurcating phantom. (E) Super-resolved image for the trifurcating phantom. (F) Velocity map of the trifurcating phantom at a flow rate of 0.02 mL/min. All scale bars are 1 mm.

Lastly, we tested the ability to fabricate complex microvasculature phantoms by creating two trifurcating phantoms. One has a main channel that splits into three channels at equal 30° angles and converges back into one channel (Figure 7A–C), and the second one has a trifurcation that splits equally at 30° angles similarly to the bifurcating phantoms (Figure 7D–F). The converging structure is frequently seen in microfluidic chips and can also mimic the capillary network architecture in vivo. Super-resolved images and velocity maps were reconstructed for both phantoms. In the velocity maps, we see a faster velocity in the main channel before the trifurcation and then a drop when the channel splits into three (Figure 7C,F). In the converging phantom, we see a slightly higher velocity in the main channel, while in the trifurcating phantom, the velocity drops by approximately a factor of 3 after splitting off from the main channel, as is expected according to conservation of mass equations.

## Discussion and Conclusions

The aim of this study was to create a fast, simple, and cost-effective microfluidic-inspired blood-vessel-mimicking phantom and evaluate the behavior of MBs within bifurcating and trifurcating vessels for ultrasound imaging applications and specifically for ULM. The vessel mimicking phantoms provide a reproducible network in which the behavior of contrast agents and accuracy of tracking algorithms can be compared under multiple controlled conditions. Multiple studies have been conducted regarding contrast agent types^26,27^ and tracking algorithms.^3,28^ We believe that the development of complex vessel mimicking phantoms can contribute to the fast-growing field of ULM by aiding in understanding the contrast agent behavior and creating a way to systematically test new localization and tracking algorithms. These phantoms may provide an alternative to computerized flow simulations used to evaluate localization and tracking accuracy by creating shapes that are challenging to reproduce with ULM, such as the horseshoe shape.^3^ We believe these phantoms can be used to study a wide variety of applications—such as the verification of contrast-enhanced harmonic imaging, resolution-improving ultrasound algorithms, and the behavior of bubble flow in therapeutic applications.^29–40^

Gelatin was selected as a tissue-mimicking material due to it being a highly accessible and cost-effective ultrasound-compatible material with acoustic properties similar to soft tissues, as it contains mostly water. Another material often used in ultrasound-compatible phantoms is agar. We found that two semipolymerized pieces of agar are much more difficult to work with as they do not connect easily. Agar is also a more brittle material, which poses a difficulty when removing the phantom from the mold with small, delicate channels. The method is versatile and can be used to create complex channel phantoms that can aid in understating and characterizing diseases that alter flow patterns such as coronary artery disease, atherosclerosis, inflammation, Crohn’s disease, cancer, and kidney diseases.^1,31^ The phantom was validated by optimizing the MB concentration and calculating velocity through individual MB tracking. Lower MB concentration negatively affected the saturation time by producing a higher τ. At a high concentration, our tracking algorithm was unable to conduct accurate tracking of the MBs and produced inconsistent and inaccurate velocity maps, while at low and medium concentrations, we were able to reliably produce accurate velocity maps. Additionally, at a high concentration, the channel itself appears almost completely white and echogenic. Our algorithm is based off the assumption that there are clear peaks in the image representing individual MBs, whereas at the higher concentration, we are unable to see clear peaks in the raw image data. It is also evident that the saturation curves of medium and high concentrations overlap. Our algorithm uses a constraint in which the localized PSFs must be at least four pixels apart from one another to avoid incorrect localizations, thus making it difficult to localize dense bubbles. As such, the one-point spread function may include multiple MBs at any concentration. Such a scenario in the phantoms that we were imaging will yield a good super-resolution image, but the tracking algorithm will not work properly, which implies that a too high concentration is used. Certain algorithms such as sparsity or deep-learning-based algorithms may be better adept for accurately localizing high concentrations of MBs and could be used with the microfluidic-inspired phantoms developed here.^32,33^ Therefore, the concentration was optimized to ensure accuracy in all of the other experiments. Next, velocity maps for three different flow rates in phantoms with different bifurcation angles showed the expected trend of a faster velocity for a larger flow rate. Velocity in the channels drops by approximately half after the bifurcation. Additionally, the velocity profiles were parabolic, as expected for laminar flow within uniform rectangular channels. Interestingly, we see slight asymmetry in the velocity profiles. We believe this may be due to the radiation force of the ultrasound wave, which slightly pushes the bubbles away from the transducer, causing bubbles to slow down as they reach the side of the channel wall. During data acquisition, we consistently see static bubbles on the channel walls furthest from the transducer until they eventually disappear, possibly being destroyed by the force of the ultrasound waves. When a bubble is stuck in place for a number of consecutive frames, our algorithm recognizes it repeatedly and tracks it as it is standing in place. These instances lower the average velocity in the bottom part of the channel, which adds to the effect in which the velocity in the upper part of the channel appears elevated in comparison to that in the bottom part of the channel. This is apparent in all reconstructed velocity maps and raises the possibility that the flow pattern of MBs may also be affected by the pressure waves in ultrafast frame rate imaging in vivo. In this case, the bubble flow was perpendicular to the wave propagation, making the effect very visible. This effect is especially apparent in Figure 3H, possibly because this phantom has a 500 μm main channel width and the effect is more visible in the wider channel. This effect could be further studied or possibly eliminated by examining bubbles that flow perpendicular to the expected flow direction. Bubbles with specific trajectories could be filtered out during post-processing. We find the bifurcation angle of a vessel between 25 and 55° to have no significant impact on saturation time when comparing the main channel and branching channel of a phantom. We also find that within the flow rates tested, between 0.01 and 0.03 mL/min, there is no significant difference of saturation time between the main and bifurcation channels.

One of the current ULM challenges is the long acquisition times necessary to reconstruct small blood vessels. Even at ultrafast frame rates of 500 Hz, acquisition time to fully image a rat brain have been reported to take 10 min.^4^ Studies have shown how slow blood flow and small vessel size negatively affect acquisition time in rat brains, with significant difference in the reconstruction time of vessels smaller than 25 μm, vessels between 30 and 50 μm, and vessels between 70 and 100 μm.^34,35^ This may be due to a combination of factors. First, the blood flow in capillaries is much slower than in larger vessels, leading to less occurrences of microbubbles in smaller vessels.^36^ Additionally, the size of MBs (average diameter of 1.5–4 μm) is within the same order of magnitude as capillaries and red blood cells in both animals and humans, further reducing the number of bubbles to flow through capillaries over a set period of time. The platform developed here can be used to study the effect of the blood-vessel diameter on ULM imaging. Our results show that when a small vessel with a diameter of 100 μm branches off a larger vessel with a diameter of 300 μm, the saturation time of the small vessel is significantly longer than the larger vessel by an average increase of 72%. We also find that in a phantom with a main channel of 500 μm and a branching channel of 200 μm, a longer saturation time is required for the smaller branching channel with an average increase of 90%.

The ability to add background scatterers to the phantoms may be relevant for many studies and allows better testing of an algorithm’s ability to distinguish microbubbles in vivo. We tested our algorithm using background scatterers and compared data acquired by using a CPS pulse sequence and a B-mode pulse sequence. In both cases, our algorithm was able to successfully remove background scatter and localize bubbles within the channel itself, although the results are slightly noisier. The main advantage of using a CPS pulse sequence lies in the ability to reduce background tissue signal and emphasize the nonlinear MB echoes.^23,37^ Many studies use simple B-mode plane wave imaging to acquire data at maximal frame rates or with additional coherently compounded angles, while CPS imaging yields a higher-quality image at a reduced frame rate. A clear difference in the contrast between the main channel and the background can be seen in the CPS images, while B-mode data often require heavier filtering to remove the background scatter.

The two additional trifurcating phantoms are shown and intended to confirm the robustness of the phantom fabrication method. The phantom that splits and converges back into one channel is especially interesting. This type of configuration is similar to those of many microfluidic PDMS devices that are readily available. A benefit of this phantom configuration is the lack of multiple outlets, which require equalization of the water pressure to ensure even flow throughout channels. Such ultrasound-compatible converging phantom cannot be fabricated using recently reported techniques.^13,9,10^ In the velocity maps, we see that the measured velocity in the converging phantom is higher in the middle channel of the trifurcation compared to that in the two side channels. This may be due to MB dynamics moving through sharper curves and hitting the phantom walls. In the trifurcating phantom that does not converge, we see better agreement in the velocity within the three channels. Additionally, we notice that the channel closest to the transducer surface in the reconstructed super-resolution image in Figure 7B appears thinner than the bottom channel. This may be due to radiation force destroying or pushing the bubbles away from the transducer toward the opposite side of the channel. This is especially noticeable in the microfluidic phantom and we see the difference in the effect of the radiation force on the bubbles in the upper channel as opposed to the lower channel, which is further from the transducer.

Regarding the study limitations, in this study, the effect of the channel size on MB behavior was examined. In vivo, red blood cells greatly outnumber MBs in the bloodstream (approximate concentration of 10^13^) and therefore may also affect the MB penetration into smaller blood vessels. Therefore, future experiments can be carried out with blood to estimate its effect.^38^ A cross-linking agent could be added to gelatin that would allow the phantoms to be maintained at above 37 °C, which would enable the study of MBs under physiological conditions. In addition, each specific microvascular network mold was fabricated using a CNC machine and cannot be altered. Here, we focused on bifurcations and trifurcations stemming from a single channel that is commonly seen in vivo, but different structures such as *Y* shapes and nonlinear channels could be studied. The smallest bifurcation angle of the phantom was limited to 25° due to technical considerations of the CNC drill used. Using our method of fabrication and the available CNC machine, we were limited to creating aluminum molds that produce channels of 100 μm width. ULM is also used to image capillaries as small as 10 μm. Photolithography presents a potential solution for enhancing mold capabilities but also raises questions about the structural integrity of gelatin within our phantom configuration when accommodating smaller channel sizes, necessitating further evaluation. Additionally, photolithography could serve to create capillary structures more closely resembling natural blood vessels, with the added complexity of aligning two semicircular sections, as explained in tissue-engineering studies.^17^ It is important to note that our current fabrication approach allows for design freedom solely within a single plane, limiting us to planar geometries.

Additionally, the inlets and outlets of the phantom are created from gelatin, which is an elastic material that changes the shape under pressure. The pressure from the tubing may change the size of the inlet over time and cause a slight leakage of the MB solution. This leakage may affect the flow rate, making it difficult to know the ground truth with complete certainty. This could be addressed by creating more tightly sealed inlets by bonding the hydrogel to glass as suggested in ref (13). Lastly, we find that there is a tendency for MBs to get stuck on the channel wall furthest from the US transducer due to the radiation force of the pressure wave. The PSF of these static bubbles may block out the PSF of flowing bubbles, creating an added challenge to tracking bubbles. We find this to have more of an effect on small channel diameters, where the width of the PSF may be larger than the width of the channel. In conclusion, we believe that these microvascular phantoms are a robust platform for precise and controlled ULM imaging and have the potential to be utilized for many diverse aspects of ultrasound imaging.

## Data Availability

All data that support the findings of this study are included within the article (and any Supporting Information files).

## Supporting Information Available

Comparison of CPS and BMode raw image data (Video 1) (MP4)
